# Is the antidepressant efficacy of ketamine and esketamine mediated via opioid mechanisms?

**DOI:** 10.1192/j.eurpsy.2026.10157

**Published:** 2026-01-29

**Authors:** Andy Lu, Heidi Xu, Gia Han Le, Christine E. Dri, Sabrina Wong, Roger Ho, Bing Cao, Heidi Ka Ying Lo, Taeho Greg Rhee, Liyang Yin, Hernan F. Guillen-Burgos, Roger S. McIntyre

**Affiliations:** 1 Department of Psychology, University of Western Ontario, London, ON, Canada; 2 Brain and Cognition Discovery Foundation, Toronto, ON, Canada; 3 Department of Toxicology and Neuroscience, University of Toronto, Toronto, ON, Canada; 4 Institute of Medical Science, University of Toronto, Toronto, ON, Canada; 5 Poul Hansen Family Centre for Depression, University Health Network, Toronto, ON, Canada; 6 Department of Psychological Medicine, Yong Loo Lin School of Medicine, National University of Singapore, Singapore; 7 Institute for Health Innovation and Technology (iHealthtech), National University of Singapore, Singapore; 8 Division of Life Science (LIFS), Hong Kong University of Science and Technology (HKUST), Clear Water Bay, Hong Kong.; 9 Key Laboratory of Cognition and Personality, Faculty of Psychology, Ministry of Education, Southwest University, Chongqing, 400715, P. R. China; 10 Department of Psychiatry, School of Clinical Medicine, LKS Faculty of Medicine, the University of Hong Kong, Hong Kong; 11 Department of Psychiatry, Yale School of Medicine, New Haven, CT, USA; 12 Department of Public Health Sciences, University of Connecticut School of Medicine, Farmington, CT, USA; 13 Pontificia Universidad Javeriana, Department of Psychiatry and Mental Health, Bogota DC, Colombia; 14 Center for Clinical and Translational Research, Bogota DC, Colombia; 15 Universidad Simón Bolívar, Center for Clinical and Translational Research, Barranquilla, Colombia; 16 Department of Psychiatry, University of Toronto, Toronto, ON, Canada.; 17 Department of Pharmacology and Toxicology, University of Toronto, Toronto, ON, Canada

**Keywords:** ketamine, esketamine, receptors, opioid, depressive disorder, treatment-resistant, major

## Abstract

**Background:**

Ketamine and esketamine produce rapid and sustained antidepressant effects in persons with treatment-resistant depression (TRD). Although it is posited that these effects are largely attributed to *N*-methyl-D-aspartate receptor antagonism, the potential involvement of the opioid system remains unclear. This systematic review investigates whether ketamine and esketamine antidepressant efficacy is mediated through the opioid system.

**Methods:**

We conducted a systematic search of preclinical and clinical studies investigating the potential involvement of the opioid system in the antidepressant effects of ketamine and esketamine. Database searches on PubMed, Cochrane Library, Embase and PsycINFO occurred from inception to September 27, 2025.

**Results:**

16 studies were identified: 12 clinical (*n* = 790) and 4 preclinical studies. Clinical designs included randomized controlled trials, case reports, pre-post studies and observational cohort studies. Preclinical studies utilized animal models of depression. Only one study examined esketamine. Naltrexone (nonselective opioid antagonist) attenuated ketamine’s effects in three studies, while four reported no such effect and one reported mixed evidence. Genetic markers of opioid receptor subtypes (i.e., OPRM1 and OPRD1) were examined in three studies, but results were inconclusive, potentially due to limited evidence. Separately, opioid use was not associated with ketamine response. Few studies directly examined opioid receptor subtypes.

**Conclusions:**

The reported mixed findings suggest that the opioid system may exert a partial mediating effect of ketamine in TRD. However, given the inconsistent attenuation of ketamine’s antidepressant effects by opioid receptor antagonists, the opioid system likely functions as a context-dependent modulator rather than a primary mediator, particularly at standard antidepressant doses.

## Introduction

Major depressive disorder (MDD) is a highly prevalent and debilitating mental disorder, which is expected to be a leading contributor to global disease burden by 2030 [1–3]. Findings from extant literature indicate MDD is associated with reduced quality of life and increased economic burden [4–6].

While monoaminergic antidepressants (e.g., selective serotonin reuptake inhibitors [SSRIs] and serotonin-norepinephrine reuptake inhibitors) are first-line pharmacotherapies for persons with MDD, a substantial proportion of patients fail to achieve a clinically meaningful response [1, 7, 8]. Persons with MDD and an inadequate response to two or more antidepressants are classified as having treatment-resistant depression (TRD) [9].

Ketamine and esketamine have demonstrated rapid, robust and sustained antidepressant effects in TRD [10–12]. Ketamine and esketamine’s antidepressant mechanism of action is posited to include *N*-methyl-D-aspartate (NMDA) receptor antagonism on GABAergic interneurons, resulting in glutamatergic disinhibition and increased AMPA receptor expression and activity [13–16].

However, findings from extant literature suggest that NMDA receptor antagonism may not fully account for ketamine and esketamine’s antidepressant effects [17–19]. Emerging evidence suggests potential involvement of the opioidergic system [20, 21]. Specifically at subanesthetic doses, ketamine and esketamine nonselectively interact with mu-opioid (MORs), delta-opioid (DORs), and kappa-opioid (KORs) receptors, suggesting that their antidepressant effects may involve opioid signaling pathways [22, 23]. Furthermore, preliminary evidence suggests that the pathophysiology of depression may involve dysregulation of endogenous opioid signaling (e.g., reduced endogenous MOR availability and KOR hyperactivation) [24–26].

Herein, the present systematic review aims to comprehensively and critically evaluate available literature on whether opioid signaling may contribute to ketamine- and/or esketamine-associated clinical symptom outcomes. This area of research holds critical implications for elucidating the mechanisms underlying ketamine and/or esketamine’s antidepressant efficacy, as well as informing safety guidelines for potential abuse liability.

## Methods

### Search strategy

This review followed the 2020 Preferred Reporting Items for Systematic Review and Meta-Analyses (PRISMA) guidelines [27]. The systematic search was conducted by retrieving articles from databases, including PubMed/MEDLINE, Cochrane Library, Embase, AMED, PsycINFO and Joanna Briggs Institute (JBI) EBP databases. The search string employed for each database included terms relevant to ketamine and esketamine, depression, opioid systems/mechanisms, and human and animal studies (Supplementary Table S1). The search occurred from inception to September 27, 2025. In addition, no search filters were applied to retrieve all potentially relevant studies.

### Study selection and eligibility criteria

Covidence was used to screen studies [28]. Two independent reviewers (A.L. and H.X.) conducted title and abstract screening for relevant studies, followed by full-text screening. Only articles deemed relevant according to the eligibility criteria (Table 1) were included for data extraction and analysis. Any conflicts were resolved between the two independent reviewers. While Covidence removed duplicate studies, a few additional duplicates were manually removed.

**Table 1.** Eligibility criteria

Inclusion criteria	Primary research (i.e., randomized controlled trials [RCTs], case–control studies, observational cohort studies, and secondary analyses) Studies that investigate the potential mediation of ketamine or esketamine antidepressant efficacy through the opioid system or mechanisms Human participants with a primary diagnosis of major depressive disorder (MDD), bipolar depression (BD), treatment-resistant depression (TRD), or treatment-resistant bipolar depression (TRBD) through the Diagnostic and Statistical Manual of Mental Disorders (DSM) or International Classification of Diseases (ICD) criteria Animal subjects must be depression models before intervention Full-text articles available and in English.
Exclusion criteria	Non-primary research (i.e., literature reviews, systematic reviews, meta-analyses, posters, abstracts, guidelines, protocols, theses, books, opinion pieces, and non-academic reports) No investigation of the potential mediation of ketamine or esketamine antidepressant efficacy through opioid systems or mechanisms Investigation of only ketamine or esketamine metabolites or arketamine Human participants without a depression diagnosis Invalid animal models of depression and depression induced only after intervention Full-text is unavailable or not in English

Studies were eligible for inclusion if they reported primary research examining the potential role of opioid systems or mechanisms in mediating the antidepressant efficacy of ketamine or esketamine. Human participants must have been diagnosed with a depressive disorder (i.e., MDD, bipolar depression [BD], TRD, or treatment-resistant BD). For animal studies, a validated depression model must have been employed before the administration of ketamine or esketamine.

### Data extraction

Data extraction was conducted by two independent reviewers (A.L. and H.X.). Extracted study characteristics for clinical/human studies were established a priori and included: (1) the first author and year of publication, (2) study design, (3) primary diagnosis, (4) total sample size (included in relevant analyses), (5) control, (6) dosage with sample size, (7) dosing frequency, (8) measure(s) (for depression), and (9) antidepressant efficacy outcomes (Table 2). Similar characteristics were extracted for preclinical/animal studies; the type of model, behavioral outcomes, and brain slice findings were extracted (Table 3).

**Table 2.** Characteristics of clinical studies investigating the potential mediating effect of the opioid system on antidepressant efficacy of ketamine or esketamine in persons with depressive disorders (*n* = 12)

Author(s)	Study design	Primary diagnosis	Total sample size	Control	Dosage (sample size)	Dosing frequency	Measure(s)	Antidepressant efficacy outcomes
**Ketamine**								
Grunebaum et al. [43]	Double-blind RCT	MDD with suicidal ideation	71	MDZ	IV ketamine infusion (0.5 mg/kg) (*n* = NR) IV MDZ infusion (0.02 mg/kg) (*n* = NR)	Ketamine ×1 infusion over 40 min MDZ ×1 infusion over 40 min	SSI POMS HDRS–17 HDRS–24	SSI, POMS, and HDRS were assessed 24 h post-infusion. Genotyping was conducted for the *OPRM1* gene. Genetic analyses revealed that 15 participants had the A118G SNP. There was no significant interaction between OPRM1 genotype (A118G SNP vs. not) and treatment (ketamine vs. MDZ). SSI Genotype × Treatment: *t*(66) = 0.59, *p* = 0.554 POMS Genotype × Treatment: *t*(64) = 0.61, *p* = 0.544 HDRS–17 Genotype × Treatment: *t*(66) = 0.78, *p* = 0.439 HDRS–24 Genotype × Treatment: *t*(66) = 0.55, *p* = 0.582 Conclusion: The OPRM1 A118G SNP did not moderate ketamine’s antidepressant efficacy in MDD (with suicidal ideation).
Hosanagar et al. [46]	Case report	TRD with suicidal ideation	1	N/A	IV ketamine infusion (0.5 mg/kg) (*n* = 1) Oral BUP (8 mg) (*n* = 1)	Ketamine ×2 infusion over 40 min weekly → 4 weeks 4 tapering infusions BUP ×3 per day	MADRS	MADRS evaluations were conducted before each ketamine treatment. Before 2nd infusion The patient reported rapid improvement in suicidality and depressive symptoms, as assessed by a moderate decrease in total MADRS score and robust decrease in MADRS suicide score. Before 3rd infusion The patient reported that their mood “markedly improved” and felt “like a fog had been lifted.” The patient lacked suicidal thoughts, as evidenced with an extremely low MADRS suicide score. They were able to perform home chores for the first time in 9 months. Post-treatment (4 weeks after the last infusion) Mood improvements were sustained and the patient did not have any suicidal ideation. Conclusion: BUP did not attenuate ketamine’s antidepressant efficacy in TRD with suicidal ideation.
Jelen et al. [39]	Double-blind crossover RCT	MDD	26	Placebo (ascorbic acid)	Oral NTX (50 mg) + IV ketamine infusion (0.5 mg/kg) (*n* = 26) Oral placebo (50 mg) + IV ketamine infusion (0.5 mg/kg) (*n* = 26)	Ketamine ×1 infusion over 40 min	MADRS QIDS-SR M3VAS SHAPS TEPS H-fMRS	Post-infusion in all participants (*n* = 26) Day 1: In the placebo + ketamine condition and NTX + ketamine condition, a significant reduction was observed in: MADRS scores (*M* = −14.65, SD = 7.77) (*M* = −10.50, SD = 5.91) (*F*_1,74_ = 197.93, *p* < 0.001). The antidepressant effect in the NTX + ketamine condition was significantly attenuated compared to the placebo + ketamine condition (MD = −4.15, SD = 8.59; *F*_1,74_ = 5.39, *p* = 0.023; Cohen’s *d* = 0.60). QIDS-SR scores (*M* = −6.54, SD = 5.43) (*M* = −5.73, SD = 3.62) (*F*_1,74_ = 96.43, *p* < 0.001). There was no significant difference in antidepressant effect between conditions (*F*_1,74_ = 0.42, *p* = 0.520). M3VAS scores (*M* = −55.59, SD = 57.12) (*M* = −45.27, SD = 46.96) (*F*_1,74_ = 47.98, *p* < 0.001). There was no significant difference in antidepressant effect between conditions (*F*_1,74_ = 0.50, *p* = 0.484). SHAPS scores (*M* = −4.00, SD = 7.47) (*M* = −2.00, SD = 4.96) (*F*_1,74_ = 12.87, *p* = 0.006) There was no significant difference in antidepressant effect between conditions (*F*_1,74_ = 1.43, *p* = 0.236) TEPS-A scores (*M* = 4.07, SD = 6.52) (*M* = 2.08, SD = 7.41) (*F*_1,74_ = 8.84, *p* = 0.004) There was no significant difference in antidepressant effect between conditions (*F*_1,74_ = 0.93, *p* = 0.338) TEPS-C scores (*M* = 2.42, SD = 5.73) (*M* = 1.69, SD = 4.35) (*F*_1,74_ = 7.89, *p* = 0.006) There was no significant difference in antidepressant effect between conditions (*F*_1,74_ = 0.25, *p* = 0.619) Post-infusion in ketamine responders Day 1: In the placebo + ketamine condition and NTX + ketamine condition, a significant reduction was observed in: MADRS scores (*n* = 14) (*M* = −20.43, SD = 3.92) (*M* = −12.00, SD = 6.62) (*F*_1,38_ = 226.90, *p* < 0.001) The antidepressant effect in the NTX + ketamine condition was significantly attenuated compared to the placebo + ketamine condition (MD = 8.43, SD = 8.28; *F*_1,38_ = 15.33, *p* < 0.001; Cohen’s *d* = 1.56). QIDS-SR scores (*n* = 13) (*M* = −10.15, SD = 3.60) (*M* = −4.62, SD = 3.82) (*F*_1,35_ = 74.76, *p* < 0.001) The antidepressant effect in the NTX + ketamine condition was significantly attenuated compared to the placebo + ketamine condition (MD = 5.54, SD = 5.70; *F*_1,38_ = 10.51, *p* = 0.003; Cohen’s *d* = 1.49). Other analyses with clinical measures On day 1, the effect of treatment conditions on clinical measures did not differ across time, between sexes, or antidepressant status (all *p* > 0.05). There was no significant difference in antidepressant effects between conditions across days 1, 3, and 7 on self-reported clinical measures, including the QIDS-SR, M3VAS, SHAPS, TEPS-A, and TEPS-C. Between conditions, there was often no significant difference in response rate and remission rate: MADRS Day 1: Response: *p* = 0.089 (almost significant) Remission: *p* = 0.034 QIDS-SR Day 1: Response: *p* = 0.400 Remission: *p* = 0.258 Day 3: Response: *p* = 0.404 Remission: *p* = 0.393 Day 7: Response: *p* = 1.000 Remission: *p* = 1.000 H-fMRS (Glx/tNAA ratio in ACC) (*n* = 24) H-fMRS was measured over 6 ketamine infusion blocks (5 min per block for 30 min total). Glx/tNAA increase in the NTX + ketamine condition was significantly attenuated compared to the placebo + ketamine condition (*F*_1,253_ = 4.83, *p* = 0.029, Cohen’s *d* = 0.34). Glx/tNAA pattern of change across infusion blocks did not significantly differ between conditions (*F*_5,253_ = 1.29, *p* = 0.270) After adjusting for age, sex, gray/white-matter fraction, SNR, antidepressant status, and pretreatment order: Difference in Glx/tNAA increase between conditions remained significant (all *p* < 0.05). Larger placebo-naltrexone difference in males (*F*_1,242_ = 4.81, *p* = 0.029). Larger placebo-naltrexone difference when naltrexone, then placebo order was used (*F*_1,242_ = 4.22, *p* = 0.041). No significant difference across age, gray/white-matter fraction, SNR, or antidepressant status. Conclusion: There is mixed evidence that NTX attenuated ketamine’s antidepressant efficacy in MDD.
Kao et al. [42]	Double-blind RCT	TRD	65	Placebo (saline)	IV ketamine infusion (0.5 mg/kg) (*n* = 21) IV ketamine infusion (0.2 mg/kg) (*n* = 20) IV placebo saline infusion (*n* = 24)	Ketamine ×1 infusion over 40 min	HDRS MADRS	HDRS and MADRS were evaluated 40- and 240-min post-infusion, and on days 2, 3, 7, and 14 post-infusion. A 50% reduction in scores indicated clinical response to ketamine (both ketamine conditions were combined in analysis). Genotyping was conducted for all participants, including analysis of the *OPRM1*, *OPRK1*, and *OPRD1* genes. All *p*-values were corrected for multiple testing corrections.SNP-based GWAS Associations Two SNPs in OPRM1 (rs2473546 and rs9479827) were significantly associated with antidepressant response to low-dose ketamine on days 3 and 14 post-infusion. rs2473546 (Day 3) HDRS Decreasing: *p* = NS Response: OR = 4.07, *p* = 6.9 × 10^−3^ MADRS Decreasing: *p* = NS Response: *p* = NS rs62432719 (Day 14) HDRS Decreasing: *p* = NS Response: *p* = NS MADRS Decreasing: *p* = NS Response: OR = 6.03, *p* = 8.3 × 10^−3^ Gene-based GWAS results Significant association between OPRD1 and antidepressant efficacy after 240 min and day 2 post-infusion. OPRD1 (240 min) HDRS Decreasing: *p* = 4.6 × 10^−2^ Response: *p* = NS MADRS Decreasing: *p* = 4.7 × 10^−2^ Response: *p* = NS OPRD1 (Day 2) HDRS Decreasing: *p* = 4.9 × 10^−2^ Response: *p* = NS MADRS Decreasing: *p* = NS Response: *p* = NS Significant association between OPRM1 and antidepressant response on days 3 and 14. OPRM1 (Day 3) HDRS Decreasing: *p* = NS Response: *p* = 3.3 × 10^−2^ MADRS Decreasing: *p* = NS Response: *p* = NS OPRM1 (Day 14) HDRS Decreasing: *p* = NS Response: *p* = NS MADRS Decreasing: *p* = 2.6 × 10^−2^ Response: *p* = NS Conclusion: The *OPRM1* and *OPRD1* genes were associated with ketamine’s antidepressant efficacy in TRD. The OPRM1 SNPS – rs2473546 and rs62432719 specifically – were associated with ketamine’s antidepressant efficacy.
Lii et al. [40]	Double-blind RCT	MDD	40	Placebo (saline)	IV ketamine infusion (0.5 mg/kg) (*n* = 20) IV placebo saline infusion (*n* = 20)	Ketamine ×1 infusion over 40 min	MADRS HADS	MADRS and HADS scores were obtained on days 1, 2, 3, 5, 7, and 14 after surgery and administration of ketamine. Ketamine Baseline opioid use status did not significantly predict post-treatment MADRS scores. Group: *t* = 0.68, *p* = 0.497 Time: *t* = 1.52, *p* = 0.127 Interaction: *t* = −0.40, *p* = 0.686 Baseline opioid use status did not significantly predict post-treatment % change in MADRS scores. Group: *t* = 0.20, *p* = 0.840 Time: *t* = 0.55, *p* = 0.579 Interaction: *t* = 0.25, *p* = 0.806 Baseline opioid use status did not significantly predict post-treatment HADS scores. Group: *t* = −0.11, *p* = 0.916 Time: *t* = −1.55, *p* = 0.122 Interaction: *t* = 1.15, *p* = 0.251 Baseline opioid use status did not significantly predict post-treatment % change in HADS scores. Group: *t* = 0.23, *p* = 0.814 Time: *t* = −1.66, *p* = 0.097 Interaction: *t* = 0.85, *p* = 0.397 Postoperative opioid use status did not significantly predict post-treatment MADRS scores. Group: *t* = −0.35, *p* = 0.728 Time: *t* = 1.18, *p* = 0.237 Interaction: *t* = −0.23, *p* = 0.819 Postoperative opioid use status did not significantly predict post-treatment HADS scores. Group: *t* = −0.22, *p* = 0.826 Time: *t* = −0.91, *p* = 0.364 Interaction: *t* = 0.24, *p* = 0.814 Conclusion: Baseline and postoperative opioid use status was not associated with ketamine’s antidepressant efficacy in MDD. However, this may be due to ketamine’s ability to reverse opioid tolerance.
Marton et al. [45]	Retrospective observational cohort study	TRD	40	N/A	IV ketamine infusion (0.5 mg/kg) (*n* = 40) Oral BUP (2–24 mg) (*n* = 5) Oral MTD (62/160 mg) (*n* = 2) IM NTX injection (380 mg) (*n* = 1)	Ketamine ×2 infusion over 40 min weekly → 3 weeks BUP ×1 daily MTD ×1 daily NTX ×1 injection every 4 weeks	BDI-II	BDI-II evaluations were conducted prior to each ketamine infusion. MOR agonist group (*n* = 7) received BUP or MTD, while the non-MOR agonist group (*n* = 27) received no opioid-interacting agents. Linear mixed model revealed significant reductions in BDI-II scores over the 6 infusions (time effect: *p* < 0.001) across all participants receiving ketamine. Average change in BDI-II scores (pre- to post-treatment) MOR group: 41 → 16 = −25 mean reduction Non-MOR group: 34 → 16 = −18 mean reduction NTX patient: 40 → 19 = −21 mean reduction No significant difference was observed between the MOR agonist and non-MOR agonist groups, pre- (group effect: *p* = 0.82) and post- (group × time: *p* = 0.11) treatment. The single patient receiving NTX exhibited an antidepressant response to ketamine comparable to those observed in the larger patient cohorts. Conclusion: BUP, MTD, and NTX did not attenuate ketamine’s antidepressant efficacy in TRD.
Quintanilla et al. [41]	Double-blind crossover RCT	MDD	64	Placebo (saline)	IV ketamine infusion (0.5 mg/kg) (*n* = 64) IV placebo saline infusion (*n* = 64)	Ketamine ×1 infusion over 40 min	MADRS SHAPS TEPS	MADRS, SHAPS, and TEPS assessments occurred at 1 h before infusion, 40-, 80-, 120-, and 230-min post-infusion, and days 1, 2, 3, 7, 10, and 11 post-infusion. Blood samples were obtained at baseline, 230 min, day 1, and day 3 post-infusion. Plasma concentrations of soluble KORs and dynorphins were quantified using commercial ELISA kits. Baseline in MDD ± HC MDD group (*n* = 39) had significantly lower KOR levels than the HC group (*n* = 25) (*F*_1,60_ = 0.13, *p* < 0.001). No significant difference was observed in dynorphin levels between groups (*F*_1,59_ = 1.67, *p* = 0.20). Post-infusion in MDD Ketamine was not significantly associated with post-infusion changes in KOR and dynorphin levels after controlling for order of infusion, age, and sex. KOR levels: Drug: *F*_1,32_ = 3.45, *p* = 0.07 Drug × Time: *F*_2,67_ = 0.97, *p* = 0.38 Dynorphin levels: Drug: *F*_1,55_ = 0.78, *p* = 0.38 Drug × Time: *F*_2,132_ = 0.22, *p* = 0.81 KOR levels as a moderator in MDD Baseline KOR levels did not moderate ketamine’s effect on depressive and anhedonia symptoms before or after controlling for multiple comparisons. Interactions were controlled for order of infusion, age, sex, and baseline KOR levels. MADRS (total scale) Drug × Biomarker: *F* = 0.33, *p* = 0.57 Drug × Biomarker × Time: *F* = 0.36, *p* = 0.70 SHAPS (total scale) Drug × Biomarker: *F* = 0.04, *p* = 0.85 Drug × Biomarker × Time: *F* = 0.02, *p* = 0.98 TEPS (total score) Drug × Biomarker: *F* = 0.43, *p* = 0.52 Drug × Biomarker × Time: *F* = 0.60, *p* = 0.55 TEPS-A Drug × Biomarker: *F* = 0.39, *p* = 0.54 Drug × Biomarker × Time: *F* = 0.23, *p* = 0.79 TEPS-C Drug × Biomarker: *F* = 0.30, *p* = 0.59 Drug × Biomarker × Time: *F* = 1.27, *p* = 0.29 Dynorphin levels as a moderator in MDD Uncorrected analyses suggested that after ketamine (but not after placebo), higher baseline dynorphin levels were associated with greater reduction in MADRS scores (*F*_1,38_ = 7.40, *p* = 0.01). However, this interaction became nonsignificant after sensitivity analyses and remained nonsignificant after correcting multiple comparisons. Interactions were controlled for order of infusion, age, sex, and baseline dynorphin levels. MADRS (total scale) Drug × Biomarker: *F* = 2.85, *p* = 0.10 Drug × Biomarker × Time: *F* = 1.19, *p* = 0.31 SHAPS (total scale) Drug × Biomarker: *F* = 0.23, *p* = 0.64 Drug × Biomarker × Time: *F* = 0.48, *p* = 0.62 TEPS (total score) Drug × Biomarker: *F* = 0.08, *p* = 0.78 Drug × Biomarker × Time: *F* = 1.10, *p* = 0.34 TEPS-A Drug × Biomarker: *F* = 0.04, *p* = 0.84 Drug × Biomarker × Time: *F* = 1.28, *p* = 0.29 TEPS-C Drug × Biomarker: *F* = 0.05, *p* = 0.83 Drug × Biomarker × Time: *F* = 1.08, *p* = 0.35 Conclusion: KOR and dynorphin plasma levels did not moderate the association between ketamine and reductions in depressive and anhedonic symptoms in MDD. Ketamine was not associated with changes in KOR and dynorphin levels.
Williams et al. [37]	Double-blind crossover RCT	TRD	14	Placebo (oral)	Oral NTX (50 mg) + IV ketamine infusion (0.5 mg/kg) (*n* = 12) Oral placebo + IV ketamine infusion (0.5 mg/kg) (*n* = 12) Two participants discontinued. One is because of adverse events, and the other is due to a need for increased care.	Ketamine ×1 infusion over 40 min	HDRS–17 (item 3) MADRS (item 10) C-SSRS	Post-infusion in ketamine responders (*n* = 7) Day 1, 3, 5, 7, 14: In the placebo + ketamine condition, a significantly greater reduction in HDRS–17 (item 3) scores was observed compared to the NTX + ketamine condition on day 1 (*p* < 0.001), day 3 (*p* = 0.014), day 5 (*p* = 0.019), day 7 (*p* < 0.001), and day 14 (*p* = 0.003). A similar difference was observed in MADRS (item 10) scores on day 1 (*p* < 0.001), day 3 (*p* = 0.002), day 5 (*p* = 0.002), day 7 (*p* < 0.001), and day 14 (*p* < 0.001). Day 3, 7, 14: For C-SSRS scores, a significant difference was observed only on day 3 (*p* = 0.002), day 7 (*p* = 0.006), and day 14 (*p* = 0.004). Post-infusion in all participants (*n* = 12) HDRS–17 (item 3) Significant effects of treatment condition (*F*_1,107_ = 7.399, *p* = 0.008), day (*F*_5,107_ = 9.014, *p* < 0.001), and day × treatment × responder (*F*_16,107_ = 3.165, *p* < 0.001). No significant effect on responder status. MADRS (item 10) Significant effects of treatment condition (*F*_1,106_) = 8.825, *p* = 0.004), day (*F*_5,106_ = 7.968, *p* < 0.001), responder status (*F*_1,10_ = 6.140, *p* = 0.032), and day × treatment × responder (*F*_16,106_ = 4.412, *p* < 0.001). C-SSRS Significant effect of treatment condition (*F*_1,104_ = 12.627, *p* = 0.001). No significant effect for day, responder status, or day × treatment × responder. Conclusion: NTX attenuated ketamine’s anti-suicidal effects in TRD.
Williams et al. [23]	Double-blind crossover RCT	TRD	14	Placebo (oral)	Oral NTX (50 mg) + IV ketamine infusion (0.5 mg/kg) (*n* = 12) Oral placebo + IV ketamine infusion (0.5 mg/kg) (*n* = 12) Two participants discontinued. One is because of adverse events, and the other is due to a need for increased care.	Ketamine ×1 infusion over 40 min	HAM-D (17-item +6-item) MADRS BDI-II	Post-infusion in ketamine responders (*n* = 7) Day 1: In the placebo + ketamine condition and NTX + ketamine condition, a significant reduction was observed in 17-item HAM-D scores (*M* = −22.3, SD = 3.2; *F* = 106, *p* < 0.001) (*M* = −5.6, SD = 5.7; *F* = 6.8, *p* = 0.04). The antidepressant effect in the NTX + ketamine condition was significantly attenuated compared to the placebo + ketamine condition (MD = −16.7, SD = 6.7; *F* = 43.7, *p* < 0.001; Cohen’s *d* = 2.5). In the placebo + ketamine condition, a significant reduction was observed in 6-item HAM-D scores (*M* = −11.7, SD = 3.1; *F* = 93.8, *p* < 0.001). However, in the NTX + ketamine condition, a nonsignificant reduction was observed (*M* = −2.4, SD = 2.8; *F* = 5.4, *p* = 0.059). In addition, the reduction in 6-item HAM-D scores in the NTX + ketamine condition was significantly lower compared to the placebo + ketamine condition (MD = 9.3, SD = 4; *F* = 29.8, *p* = 0.002; Cohen’s *d* = 2.3). After day 1, five of seven responders in the placebo + ketamine condition met criteria for remission according to the 17-item HAM-D. Comparatively, none of the seven responders in the NTX + ketamine condition met criteria for remission. Day 3: A significant difference in scores between the two conditions continued. Days 5, 7, 14: No significant difference in scores was observed. Post-infusion in all participants (*n* = 12) Day 1: A significant reduction in 17-item HAM-D scores was observed in both conditions (placebo + ketamine (*M* = −14.2, SD = 10.7; *F* = 8.7, *p* = 0.013) and NTX + ketamine (*M* = −4.9, SD = 6.8; *F* = 8.7, *p* = 0.013)). Notably, the reduction was smaller in the NTX + ketamine condition (MD = −8.4, SD = 12.6; condition-by-time interaction, *F* = 5.4, *p* = 0.041; Cohen’s *d* = 0.7). A significant reduction in 6-item HAM-D scores was observed in the placebo + ketamine condition (*M* = −7.5, SD = 5.8). However, the reduction was nonsignificant in the NTX + ketamine condition (*M* = −2.0, SD = 3.9; *F* = 3.0, *p* = 0.11). Notably, the reduction was significantly attenuated (MD = 5.5, SD = 6.9; *F* = 7.7, *p* = 0.018; Cohen’s *d* = 0.8). MADRS and BDI-II Ketamine’s effect on MADRS and BDI-II scores was significantly reduced by NTX in ketamine responders. Similar to the 6-item HAM-D scores, MADRS scores did not show a significant reduction from baseline to post-infusion day 1 in the NTX + ketamine group. Conclusion: NTX attenuated ketamine’s antidepressant efficacy in TRD.
Yoon et al. [38]	Double-blind RCT	MDD	58	Saline	IM NTX injection (380 mg) + IV ketamine infusion (0.5 mg/kg) (*n* = 20) IM saline injection + IV ketamine infusion (0.5 mg/kg) (*n* = 19) IM saline injection + IV MDZ infusion (0.045 mg/kg) (*n* = 19)	Ketamine ×1 infusion over 40 min weekly → 4 weeks NTX ×1 injection before the first ketamine infusion MDZ ×1 infusion over 40 min weekly → 4 weeks	MADRS	Clinical response (≥50% reduction in MADRS scores) was assessed 60 min before and 240 min after each infusion. Clinical response was assessed until the primary end-of-treatment time point, 240 min after the final ketamine infusion (day 21, visit 6, week 3). Clinical response Clinical response was comparable across conditions at primary end-of-treatment time point (NTX-ketamine [93.8%], saline-ketamine [81.8%], saline-MDZ [86.7%]; *p* = 0.63). There were no significant differences in clinical response between conditions at any assessment conducted 60 min before infusion. MADRS scores MADRS scores were significantly reduced across assessments 60 min before infusion (*F*_5,214_ = 34.0, *p* < 0.0001) and 240 min after infusion (*F*_5,213_ = 172.6, *p* < 0.0001). There were no significant differences in MADRS scores between conditions at any assessment conducted 240 min after infusion (*F*_10,213_ = 1.79, *p* = 0.06). Note, this finding was almost significant. There were no significant differences in MADRS scores between conditions at any assessment conducted 60 min before infusion (*F*_10,214_ = 0.77, *p* = 0.66). However, in week 4 (no infusion administered), MADRS scores were significantly lower in the NTX-ketamine (*t*_213_ = −2.25, *p* = 0.026) and saline-ketamine (*t*_213_ = −2.11, *p* = 0.036) conditions compared to saline-MDZ. Conclusion: NTX did not attenuate ketamine’s antidepressant efficacy in MDD.
Yoon et al. [47]	Prospective open-label study	MDD	5	N/A	IV ketamine infusion (0.5 mg/kg) (*n* = 5) IM NTX injection (380 mg) (*n* = 5)	Ketamine ×1 infusion weekly → 4 weeks NTX ×1 injection 2–6 days before the first ketamine infusion	MADRS	MADRS evaluations were conducted 4 h after each post-infusion. NTX + ketamine was associated with reduced depressive symptoms. First infusion: 60% (*n* = 3) achieved 50% + reduction in MADRS scores (clinical response). Fourth infusion: 100% (*n* = 5) achieved clinical response. From baseline to post-treatment of final infusion, depressive symptoms (MADRS scores) improved 57–92% across all participants. Conclusion: NTX did not attenuate ketamine’s antidepressant efficacy in MDD.
**Esketamine**								
Saad et al. [44]	Double-blind RCT	TRD	406	Placebo (nasal spray) with oral antidepressant	IN esketamine + Oral antidepressant (84 mg) (*n* = 233) Placebo nasal spray + Oral antidepressant (*n* = 173) Oral antidepressant: Escitalopram, Sertraline, Duloxetine, or Venlafaxine XR)	2× spray weekly → 4 weeks	MADRS	Esketamine + Antidepressant The OPRM1 SNP rs1799971 was not significantly associated with reductions in MADRS scores on days 2 and 28. Day 2: *p* = 0.69, *R* ^2^_partial_ = < 0.5% Day 28: *p* = 0.34, *R* ^2^_partial_ = < 0.5% No significant associations were found for the OPRM1 SNP rs34427887 as well. Conclusion: The OPRM1 SNPs rs1799971 and rs34427887 were not associated with esketamine’s antidepressant efficacy in TRD. However, this may be because esketamine’s concentrations in the brain at therapeutic doses are far too low to significantly bind to or activate MORs.

Abbreviations: ACC, anterior cingulate cortex; BDI-II, Beck Depression Inventory-Second Edition; BUP, buprenorphine; C-SSRS, Columbia Suicide Severity Rating Scale; ELISA, enzyme-linked immunosorbent assay; Glx, combined measure of glutamate and glutamine; GWAS, genome-wide association studies; HADS, Hospital Anxiety and Depression Scale; HAM-D, Hamilton Depression Rating Scale; HC, healthy controls; HDRS, Hamilton Depression Rating Scale; H-fMRS, proton functional magnetic resonance spectroscopy; IM, intramuscularly; IN, intranasal; IV, intravenous; KOR, kappa opioid receptor; M, mean; M3VAS, Maudsley 3-item Visual Analogue Scale; MADRS, Montgomery–Åsberg Depression Rating Scale; MD, mean difference; MDD, major depressive disorder; MDZ, midazolam; MOR, mu opioid receptor; MTD, methadone; NR, not reported; NS, not significant; NTX, naltrexone; OR, odds ratio; POMS, Profile of Mood States; QIDS-SR, Quick Inventory of Depressive Symptomatology Self-Report; RCT, randomized controlled trial; SD, standard deviation; SHAPS, Snaith–Hamilton Pleasure Scale; SNP, single nucleotide polymorphism; SNR, signal-to-noise ratio; SSI, Beck Scale for Suicidal Ideation; TEPS, Temporal Experience of Pleasure Scale; TEPS-A, TEPS anticipatory subscale; TEPS-C, TEPS consummatory subscale; tNAA, *N*-acetylaspartate + *N*-acetylaspartylglutamate; TRD, treatment resistant depression.

**Table 3.** Characteristics of preclinical studies investigating the potential mediating effect of the opioid system on behavioral outcomes of ketamine in depressed animal models (*n* = 4)

Author(s)	Study design	Model	Control	Dosage	Dosing frequency	Measure(s)	Behavioral outcomes
**Ketamine**							
Klein et al. [33]	In vivo + Ex vivo (brain slice)	Male Sprague–Dawley cLH rats	Vehicle (saline)	In-vivo IP ketamine injection (15 mg/kg) SC NTX hydrochloride (1 mg/kg) Vehicle (saline) Ex-vivo Ketamine via the ACSF perfusate (10 μM) NTX (1 μM) CTAP (100 nM)	Ketamine ×1 IP injection NTX ×1 SC 1 h before ketamine	mFST PRT	mFST and PRT were measured 2 h after the ketamine injection. mFST Ketamine alone significantly reduced immobility in cLH rats. NTX + Ketamine removed ketamine’s effect on immobility; behavior resembled vehicle-treated rats. Pre-treatment (NTX) × treatment (ketamine): *F*(1,50) = 9.73, *p* < 0.005 NTX + ketamine versus vehicle + ketamine: *p* < 0.05 PRT Ketamine alone significantly increased the breakpoint in cLH rats NTX + Ketamine completely blocked this effect. Ketamine versus NTX + Ketamine: *F*(2,30) = 5.79, *p* < 0.05 LHb hyperactivity Ketamine alone acutely reduced cellular activity of LHb in cLH rats. NTX blocked the effects of ketamine, with no reduced activity of LHb. Pretreatment (NTX) × treatment (ketamine): *F*(1,702) = 11.17, *p* < 0.005 Ketamine versus NTX + Ketamine: *p* < 0.05 CTAP produced a blocking effect similar to NTX. Pretreatment (CTAP) × Treatment (ketamine): *F*(1,705) = 11.17, *p* < 0.005 Ketamine versus CTAP + Ketamine: *p* < 0.05 Conclusion: NTX and CTAP attenuated ketamine’s antidepressant efficacy in cLH rats.
Pomrenze et al. [34]	In vivo	C57BL/ 6 J mice CORT model	Placebo (saline)	IP ketamine (30 mg/kg) IP NTX (5 mg/kg) Placebo saline	Ketamine ×1 IP injection NTX ×1 IP injection 30 min before ketamine	FST	FST was measured 24 h after ketamine injection. FST 1% CORT in drinking water for 10 days. Ketamine administered 24 h before FST showed reduced immobility in both CORT and non-stressed mice. NTX did not block the antidepressant-like effect of ketamine in the CORT model CORT: Placebo versus Ketamine: *p* < 0.0001. CORT: Placebo versus NTX + Ketamine: *p* < 0.0001. Conclusion: NTX did not attenuate ketamine’s antidepressant efficacy in CORT models of mice.
Reddy et al. [36]	In vivo + Ex vivo (brain slice)	Adult zebrafish CUS model	N/A	Ketamine via immersion in a water tank (20 mg/L)	Ketamine ×1 over 15 min → 4 days in a row	SI FAT TRT NTT	CUS in zebrafish caused depressive behaviors and changes in brain protein expression, especially in the telencephalon. Ketamine reversed many depressive behaviors (as assessed by SI, FAT, TRT, and NTT) and protein changes in the span of 12 days. Using IPA, opioid signaling was found to be a key canonical pathway altered. Opioid signaling was significantly dysregulated during CUS-induced depression and normalized after ketamine treatment. Proteins: RICTOR, PER2, CABP1 RICTOR was downregulated in CUS and normalized after ketamine; it is required for opioid-induced neural plasticity. PER2 was upregulated during CUS and normalized after ketamine; it modulates MOR function. CABP1 was dysregulated in CUS and normalized after ketamine; it interacts with Ca^2+^ channels (downstream of opioid receptors). Conclusion: Ketamine’s antidepressant effect involves opioid systems, as evidenced by normalized protein expression after ketamine intervention in CUS zebrafish.
Zhang and Hashimoto [35]	In vivo	Male adult C57BL/6 + CD1 mice CSDS model LPS model	Placebo (saline)	IP ketamine injection (10 mg/kg) IP NTX injection (10 mg/kg) IP placebo saline Injection (10 mL/kg) LPS (0.5 mg/kg)	Ketamine ×1 injection NTX ×1 injection	FST TST SPT	CSDS (*n* = 40) Ketamine alone reduced depressive behaviors; decreased immobility in TST and FST, increased sucrose preference in SPT in the span of 14 days. NTX + Ketamine did not block ketamine’s antidepressant effects. FST: *F*(4,35) = 6.672, *p* < 0.001 TST: *F*(4,35) = 9.473, *p* < 0.001 SPT: *F*(4,35) = 5.146, *p* = 0.002 LPS (*n* = 40) Ketamine alone reduced depressive behavior in FST in 3–4 days. NTX + Ketamine did not prevent antidepressant response. FST: *F*(4,35) = 3.627, *p* = 0.014 Conclusion: NTX did not attenuate ketamine’s antidepressant efficacy in CSDS and LPS mice.

Abbreviations: ACSF, artificial cerebrospinal fluid; cLH, congenital learned helplessness; CORT, corticosterone; CSDS, chronic social defeat stress; CTAP, D-Phe-Cys-Tyr-D-Trp-Arg-Thr-Pen-Thr-NH2; CUS, chronic unpredictable stress; FAT, feed approach test; FST, forced swim test; IP, intraperitoneal; IPA, Ingenuity Pathway Analysis; LHb, lateral habenula; LPS, lipopolysaccharide-induced inflammation; mFST, modified forced swim test; NTT, novel tank test; NTX, naltrexone; PRT, progressive ratio task; SI, social interaction test; SPT, sucrose preference test; SC, subcutaneous; TRT, threat response test; TST, tail suspension test; WT, wild type.

### Quality assessments

Using the National Institutes of Health tools, independent reviewers (A.L. and H.X.) appraised the included clinical/human studies. In particular, this review utilized the Quality Assessment of Controlled Intervention Studies, the Quality Assessment of Observational Cohort and Cross-Sectional Studies, and the Quality Assessment Tool for Before-After (Pre-Post) Studies with No Control Group [29, 30]. In addition, the JBI Critical Appraisal Tool for Case Reports was used [29, 31]. For preclinical/animal studies, the SYRCLE’s Risk of Bias tool was utilized [32]. Any disagreements in assessments were resolved through discussion.

## Results

### Search results

**Figure 1.**
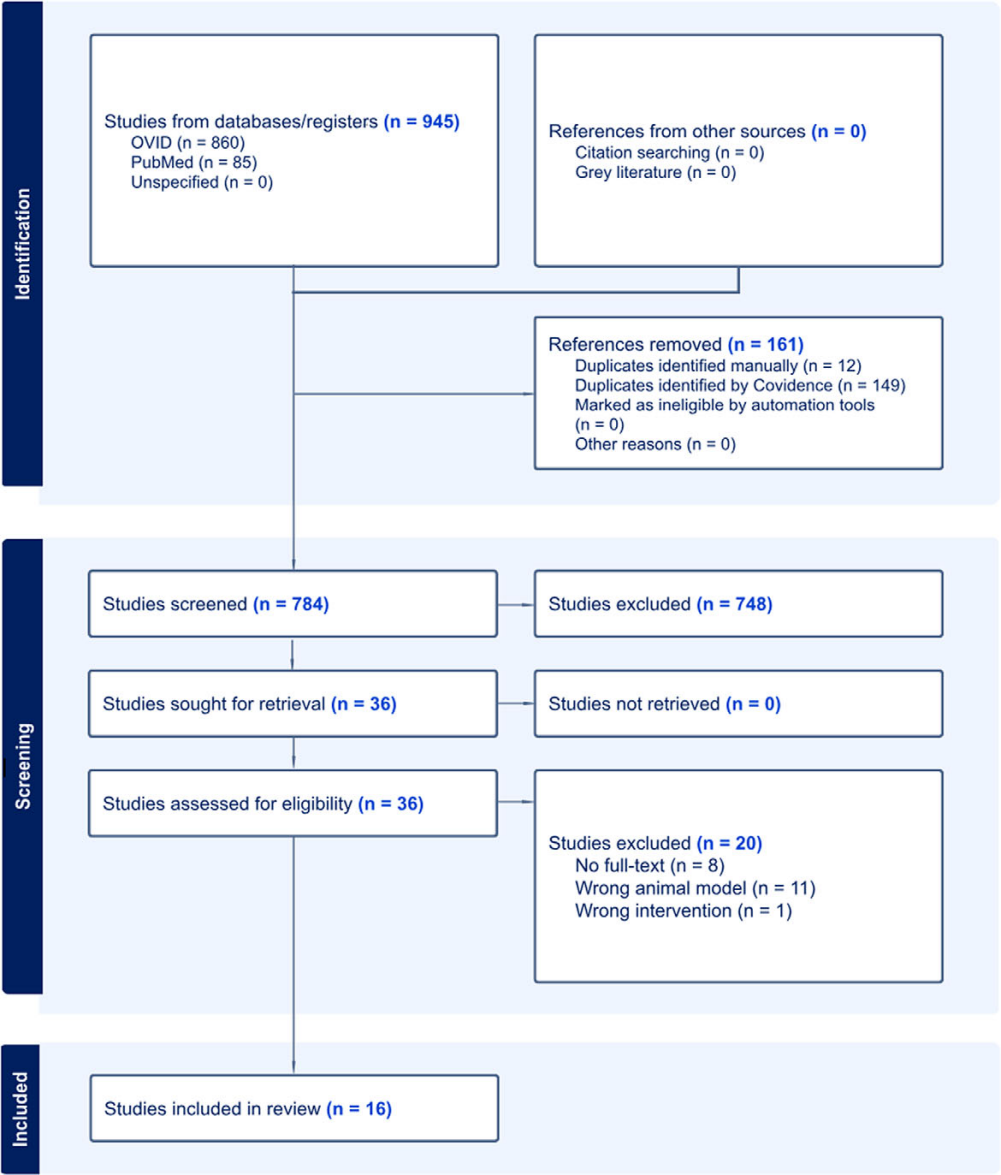
PRISMA flow diagram of literature search. *Source*: Page MJ, et al. BMJ 2021;372:n71. doi: 10.1136/bmj.n71 [28].

The search strategy yielded 784 studies after removing duplicates (*n* = 161). Using the eligibility criteria for screening (Table 1), 36 studies remained after title and abstract screening, and 16 studies were included in this review after full-text screening (*n* = 4 preclinical studies; *n* = 12 clinical studies). Reasons for excluding studies in full-text screening included the wrong animal model (*n* = 11), no full-text (*n* = 8), and the wrong intervention (*n* = 1). Other details can be found in the PRISMA flow diagram (Figure 1).

### Methodological quality assessment

Preclinical/animal studies were evaluated as a moderate risk of bias [33–36]. These evaluations were largely due to the lack of reporting of several study design details. Among clinical studies, controlled intervention studies had a low risk of bias [23, 37–44]. Similarly, Marton et al. [45] was an observational cohort study, and Hosanagar et al. [46] was a case report; they were deemed moderate and low risk of bias, respectively. In contrast, Yoon et al. [47] was a pre-post study with no control group, and the only study assessed as high in risk of bias. All assessments can be found in the Supplementary Materials (Supplementary Tables S2–S6).

### Overview of preclinical and clinical study characteristics

There were four preclinical studies that examined ketamine with animal models of depression; none of the studies examined esketamine. A subanesthetic dose of ketamine was administered either through intraperitoneal injection (i.e., 10–30 mg/kg) [33–35], artificial cerebrospinal fluid perfusate (i.e., 10 μM) [33], or immersion in a water tank (i.e., 20 mg/L) [36].

Across all 12 clinical studies (*n* = 9 controlled intervention, *n* = 1 observational cohort, *n* = 1 pre-post with no control group, and *n* = 1 case report), a total of 706 participants (*n* = 264 MDD and *n* = 526 TRD) were included. Eleven studies investigated a subanesthetic dose of intravenous (IV) ketamine (i.e., 0.2 or 0.5 mg/kg) [23, 37–43, 45, 46, 47]. Intranasal esketamine (i.e., 84 mg) was investigated in only one study [44].

### Preclinical/animal studies

#### Effect of opioid antagonists on ketamine

Naltrexone (i.e., NTX; a nonselective opioid antagonist) attenuated ketamine’s effects in congenitally learned helplessness rats (i.e., rat model of depression) [33]. Significantly greater immobility time in the forced swimming test (FST) was observed in depressed rats receiving NTX combined with ketamine compared to ketamine-placebo (*p* < 0.05) [33]. In line with the foregoing trend, ketamine significantly increased effort exerted to obtain rewards in the progressive ratio task, indexing improvements in anhedonia symptom severity; however, this effect was not observed in the NTX-ketamine group (*F* = 5.79, *p* < 0.05) [33]. Furthermore, NTX also inhibited ketamine-induced reductions in lateral habenula (LHb) activity (i.e., LHb hyperactivity is associated with depression) (*p* < 0.05) [33].

Separately, NTX did not attenuate ketamine’s antidepressant effects in stress-induced mice, wherein a nonsignificant between-group difference in immobility time was observed [34]. Comparably, in other stress-induced models, ketamine produced antidepressant-like effects across the FST (*F* = 6.672, *p* < 0.001; *F* = 3.627, *p* = 0.014), tail suspension test (TST) (*F* = 9.473, *p* < 0.001), and sucrose preference test (*F* = 5.146, *p* = 0.002), with no observed attenuation by NTX coadministration [35].

Beyond NTX, D-Phe-Cys-Tyr-D-Trp-Arg-Thr-Pen-Thr-NH2 (opioid antagonist) also inhibited ketamine-induced reductions in LHb activity (*p* < 0.05) [33].

#### Regulation of opioid signaling by ketamine

In a chronic unpredictable stress model of zebrafish, ketamine elicited improved performance in the social interaction test, feed approach test, novel tank test and TST [36]. These behavioral effects were accompanied by normalization of dysregulated opioid signaling. Ketamine restored the expression or function of opioid-related proteins, including RICTOR, PER2 and CABP1, to baseline levels [36].

### Clinical/human studies

#### Effect of NTX on ketamine

In a crossover randomized controlled trial (RCT), NTX significantly attenuated both the overall antidepressant and anti-suicidal effects of ketamine in patients with TRD (*n* = 12) [23, 37]. Among ketamine responders (*n* = 7), reductions in Hamilton Depression Rating Scale (HDRS) total scores were significantly greater when administered placebo-ketamine compared to NTX-ketamine (HDRS 17-item: *F* = 43.7, *p* < 0.001; HDRS 6-item: *F* = 29.8, *p* = 0.002) [23]. No significant difference was observed on days 5, 7 and 14 post-treatment [23]. In addition, five out of seven responders achieved remission (a score of ≤7 on the HDRS) when administered placebo-ketamine, whereas none met the criteria when administered NTX-ketamine [23]. The attenuation effect of NTX was consistent in the Montgomery-Åsberg Depression Rating Scale (MADRS), Beck Depression Inventory Second Edition (BDI-II), and Columbia Suicide Severity Rating Scale (C-SSRS) scores, and in all participants [23, 37]. Notably, among responders, reductions differed significantly between groups for HDRS suicidal ideation (SI) score (all *p* ≤ 0.02), MADRS SI score (all *p* ≤ 0.002), and C-SSRS (all *p* ≤ 0.006) [37].

Conversely, in an observational cohort study of TRD patients, a patient (*n* = 1) received NTX-ketamine and reported BDI-II scores comparable to patients (*n* = 34) receiving ketamine alongside buprenorphine (i.e., partial MOR agonist), methadone (i.e., full MOR agonist), or no MOR agent [45]. Consistent with the foregoing findings, an open-label study of patients with MDD and alcohol use disorder (AUD) (*n* = 5) received NTX-ketamine and all achieved clinical response by the fourth infusion (50% reduction in MADRS scores), demonstrating MADRS reductions ranging from 57 to 92% after 4 weeks of treatment [47]. Similarly, in an RCT study, patients with MDD and AUD (*n* = 58) received NTX-ketamine, saline-ketamine or midazolam-ketamine and showed no significant differences in clinical response (*p* = 0.63) [38]. MADRS scores were similar across conditions 60 min before (*F*_10,214_ = 0.77, *p* = 0.66) and 240 min after infusion (*F*_10,213_ = 1.79, *p* = 0.06) [38].

In contrast, a crossover RCT of patients with MDD (*n* = 26) compared NTX-ketamine with placebo-ketamine and reported mixed evidence for opioid-mediated antidepressant effects [39]. In all participants, reductions in MADRS scores significantly differed between conditions on day 1 of post-infusion (*F*_1,74_ = 5.39, *p* = 0.023) [39]. However, the foregoing finding was not replicated on days 1, 3 and 7 in self-report measures, including the Quick Inventory of Depressive Symptomatology Self-Report (QIDS-SR), Maudsley 3-item Visual Analogue Scale, Snaith Hamilton Pleasure Scale (SHAPS), and Temporal Experience of Pleasure Scale anticipatory anhedonia subscale (TEPS-A) and consummatory anhedonia subscale (TEPS-C) (all *p* > 0.05) [39]. Among ketamine responders, the attenuation effect of NTX was more robust (MADRS: *F*_1,38_ = 15.33, *p* < 0.001; QIDS-SR: *F*_1,38_ = 10.51, *p* = 0.003) [39]. Response and remission rates were generally comparable between conditions, except for a lower MADRS remission in the NTX-ketamine condition on day 1 of post-infusion (*p* = 0.034) [39]. In a neuroimaging subsample (*n* = 24) using proton functional magnetic resonance spectroscopy, Glx/tNAA (glutamate + glutamine/*N*-acetylaspartate + *N*-acetylaspartylglutamate) ratios in the anterior cingulate cortex (ACC) increased significantly during ketamine infusion, but this increase was attenuated in the NTX-ketamine condition compared with placebo-ketamine (*F*_1,253_ = 4.83, *p* = 0.029) [39].

#### Effect of opioid use on ketamine

In a case report of a patient with TRD and active SI, Hosanagar et al. [46] reported a robust decrease in MADRS total and SI score after the first and second infusion of ketamine-buprenorphine. Mood improvements were sustained, with no report of SI 4 weeks post-treatment [46]. Similarly, Marton et al. [45] did not observe a significant difference in reductions in BDI-II scores between TRD patients administered ketamine with oral buprenorphine or methadone (*n* = 7) compared to ketamine only (*n* = 27) (*p* = 0.11).

In a separate double-blind RCT study of patients with MDD (*n* = 40), baseline opioid use status did not significantly predict post-treatment MADRS scores (*t* = −0.40, *p* = 0.686), % change in MADRS scores (*t* = 0.25, *p* = 0.806), post-treatment Hospital Anxiety and Depression Scale (HADS) scores (*t* = 1.15, *p* = 0.251), and % change in HADS scores (*t* = 0.85, *p* = 0.397) [40]. In addition, postoperative opioid use status did not significantly predict post-treatment MADRS (*t* = −0.23, *p* = 0.819) and HADS scores (*t* = 0.24, *p* = 0.814) [40].

#### Molecular biomarkers: KOR and dynorphin plasma levels

In a double-blind crossover RCT of MDD patients (*n* = 64), no significant drug × time × plasma levels of soluble KORs interaction was found for MADRS (*F* = 0.36, *p* = 0.70), SHAPS (*F* = 0.02, *p* = 0.98), TEPS total score (*F* = 0.60, *p* = 0.55), and TEPS-A (*F* = 0.23, *p* = 0.79) or TEPS-C (*F* = 1.27, *p* = 0.29) [41]. In line with the foregoing trend, a nonsignificant interaction was observed for plasma levels of dynorphins in MADRS (*F* = 1.19, *p* = 0.31), SHAPS (*F* = 0.48, *p* = 0.62), TEPS total score (*F* = 1.10, *p* = 0.34), and TEPS-A (*F* = 1.28, *p* = 0.29) or TEPS-C (*F* = 1.08, *p* = 0.35) [41].

#### Ketamine and opioid gene variants

In a double-blind RCT of TRD patients (*n* = 65), Kao et al. [42] observed a significant association between the *OPRD1* gene and reductions in HDRS (*p* ≤ 0.049) and MADRS scores (*p* = 0.047) post-treatment of ketamine. In addition, the *OPRM1* gene was associated with a clinical response (50% reduction) in HDRS (*p* = 0.033) and MADRS scores (*p* = 0.026) [42]. Notably, the OPRM1 variant rs2473546 was associated with clinical response in HDRS scores (odds ratio [OR] = 4.07, *p* = 0.0069); the rs9479827 variant was also associated with clinical response in MADRS scores (OR = 6.04, *p* = 0.0083) [42].

However, in a double-blind RCT of MDD patients with active SI (*n* = 71), Grunebaum et al. [43] observed no significant interaction between the OPRM1 A118G polymorphism and ketamine response, particularly in HDRS-17 (*t* = 0.78, *p* = 0.439), HDRS-24 (*t* = 0.55, *p* = 0.582), Beck Scale for Suicidal Ideation (*t* = 0.59, *p* = 0.554), and Profile of Mood States scores (*t* = 0.61, *p* = 0.544).

#### Esketamine and opioid gene variants

In a double-blind RCT of TRD patients (*n* = 406), Saad et al. [44] observed that the OPRM1 variant rs1799971 was not significantly associated with reductions in MADRS scores on days 2 and 28 post-treatment of esketamine combined with an antidepressant (*p* = 0.69, *R^2^*_partial_ = < 0.5%; *p* = 0.34, *R^2^*_partial_ = < 0.5%). In accordance, no significant association with esketamine response was observed for the OPRM1 variant rs34427887 [44].

## Discussion

To our knowledge, this is the first systematic review to comprehensively evaluate the role of opioid signaling in the mechanisms of action of ketamine’s and esketamine’s antidepressant efficacy. Studies reported that opioid antagonists (e.g., NTX) may attenuate ketamine’s antidepressant efficacy, particularly in small, randomized crossover trials. In contrast, larger randomized trials generally found no consistent moderation by opioid-related gene expression (i.e., *OPRM1* and *OPRD1*) or plasma biomarkers (KOR and dynorphin). Separately, opioid use (e.g., buprenorphine and methadone) did not significantly alter response to ketamine. Although definitive conclusions cannot be drawn, trends across preclinical and clinical evidence support a partial mediating effect of the opioid system on ketamine’s and esketamine’s antidepressant effects in persons with TRD.

Separately, all but one study involving persons with MDD did not observe any support for a mediation effect. The foregoing distinction may be attributed to neuropathophysiological differences between TRD and MDD populations, potentially accounting for differential treatment response [9, 48–54]. In particular, TRD is associated with lower ACC GABA levels and smaller hippocampal volumes than MDD, indicating greater GABAergic dysfunction [55]. More pronounced GABAergic deficits in TRD may render ketamine’s antidepressant effects increasingly dependent on opioid-mediated mechanisms, thereby causing NTX antagonism to be consistently disruptive. In contrast, in MDD, relatively preserved GABAergic function may subserve partial antidepressant efficacy, notwithstanding opioid receptor antagonism.

Differences in the pharmacological profiles of ketamine and esketamine, as well as the hypothesized distinct roles of MOR, DOR and KOR receptors in depression pathophysiology and antidepressant response, may also contribute to the observed variability in our findings (Supplementary Table S7) [22, 23, 25]. In addition, extant literature notes that subanesthetic ketamine and esketamine doses exhibit low binding affinity for opioid receptors, which may limit receptor engagement and the detection of opioid-mediated effects [56–60]. In particular, recent mechanistic studies suggest that ketamine and its metabolites act as positive allosteric modulators of MORs at standard antidepressant doses, enhancing opioid signaling rather than directly activating the receptor [60]. The foregoing framework may reconcile our mixed clinical findings by positing that the antidepressant effects depend on individual differences in receptor availability or opioid system responsiveness, rather than direct MOR activation [60].

### Clinical implications

Our inconclusive findings warrant further investigation, with a specific focus in clarifying ketamine’s and esketamine’s antidepressant mechanisms and informing the development of novel therapeutics. However, our findings highlight several considerations. The lack of attenuation by opioid receptor antagonists suggests that receptor activation may not be essential for antidepressant response. Notwithstanding, the observed consistent attenuation in other studies involving TRD and the aforementioned factors that may underlie variability in our results, more likely suggest that the opioid system may function as a context-dependent modulator rather than a uniform mediator. The foregoing distinction underscores the need for stratified research designs that differentiate between MDD and TRD and account for individual variability in opioid receptor availability and expression, prior opioid exposure, and genetic polymorphisms.

In addition, at subanesthetic doses of IV ketamine, there is a concern for potential abuse liability [61–64]. A scoping review by Le et al. [61] included four clinical studies and reported that patients with TRD showed no compelling evidence of dependence or diversion after a single or repeated ketamine administration in a professionally controlled setting. Similarly, results from pharmacovigilance and other inquiries have not provided evidence of drug abuse, misuse, diversion or gateway activity with ketamine or esketamine when administered under clinical supervision [65–67]. The Food and Drug Administration (FDA)―mandated Risk Evaluation and Mitigation Strategy for esketamine has also likely contributed to the reduced risk profile observed [68]. However, clinical evidence remains limited, warranting further investigation [59, 62].

### Limitations and future directions

Our review was limited to studies that investigated ketamine and esketamine in persons with depressive disorders and animal models of depression. Thus, we excluded studies on arketamine or ketamine metabolites. We also excluded preclinical studies during full-text review that used nondepressed animal models, most of which reported opioid-mediated mechanisms or irrelevant outcomes [56, 59, 69–77]. Therefore, our results cannot be generalized to other human populations, animal models or glutamatergic modulators. Studies varied considerably, such as in study designs, sample sizes, routes of administration, dosage, dosing schedule, and outcome measures, limiting direct comparisons (Supplementary Tables S6–S10). Although our review included a comparable number of MDD and TRD studies, the sample was skewed toward TRD due to one large esketamine trial. However, findings remained inconclusive as it was the only esketamine study. The inclusion of only one esketamine study also limited opportunities for direct comparison with ketamine.

Further research should adopt rigorously controlled, stratified and dose-escalation RCT designs using opioid receptor-selective probes to more precisely delineate the role of the opioid system. It is also recommended that further mechanistic research investigate esketamine’s opioid interactions because of its FDA approval for depression and more widespread clinical use compared to ketamine [78, 79].

## Supporting information

10.1192/j.eurpsy.2026.10157.sm001 Lu et al. supplementary material Lu et al. supplementary material

## Data Availability

This systematic review did not generate any new data. All extracted and analyzed data are provided in the Supplementary Materials and presented within the tables and figures of this article. Data from the original studies can be accessed through their respective publications or repositories.

## Supplementary material

The supplementary material for this article can be found at http://doi.org/10.1192/j.eurpsy.2026.10157.
