# Supplementary material for: Is the antidepressant efficacy of ketamine and esketamine mediated via opioid mechanisms?
Source: Eur Psychiatry. 2026 Jan 29;69(1):e24. doi: 10.1192/j.eurpsy.2026.10157 (PMC12925689; doi:10.1192/j.eurpsy.2026.10157)
Supplement: Lu et al. supplementary material [file S0924933826101576sup001.docx]

# **SUPPLEMENTARY MATERIALS**

## **Table S1.** Search string implemented for each database.

| **Concept** | **Search String*** |
| --- | --- |
| Ketamine / Esketamine | (“Ketamine” OR “Arketamine” OR “Esketamine” or “Ketalar” or “Spravato”) |
| Depression | (“Depression” OR “Major Depressive Disorder” OR “MDD” OR “Bipolar Depression” OR “BD” OR “Treatment Resistant Bipolar Depression” OR “TRBD” OR “Treatment Resistant Depression” OR “TRD” OR “Antidepressant”) |
| Opioid Systems / Mechanisms | (“Opioid System” OR “Opioid Mechanism” OR “Opioid Receptor” OR “Opioid Signaling” OR “Opioid Pathway” OR “Opioid Activity” OR “Opioid Modulation” OR “Opioid Neurotransmission” OR “Opioid Binding” OR “Opioid-Mediated” OR “Opioid-Dependent” OR “Opioid-Related Mechanism” OR “Opioid Effects” OR “Opioid Function” OR “Mu-Opioid Receptor” OR “MOR” OR “Delta-Opioid Receptor” OR “DOR” OR “Kappa-Opioid Receptor” OR “KOR” OR “Nociceptin Receptor” OR “NOP” OR “Orphanin FQ Receptor” OR “ORL1” OR “Naltrexone” OR “Naloxone” OR “Nalmefene”) |
| Human / Animal Studies | (“Human” OR “Patient” OR “Subject” OR “Participant” OR “Clinical Trial” OR “Animal” OR “Rodent” OR “Rat” OR “Mouse” OR “Murine” OR “Preclinical” OR “Animal Experimentation”) |

*The search string for each concept was combined with the boolean operator “AND”.

##

##

##

##

## **Table S2.** Risk of bias/quality assessment of the included studies using the NIH Quality Assessment of Controlled Intervention Studies [29,30].

| **Study** | **Item** | | | | | | | | | | | | | | **Quality Rating** |
| --- | --- | --- | --- | --- | --- | --- | --- | --- | --- | --- | --- | --- | --- | --- | --- |
|  | **1** | **2** | **3** | **4** | **5** | **6** | **7** | **8** | **9** | **10** | **11** | **12** | **13** | **14** |  |
| Grunebaum et al. (2020)  [43] | ✓ | ✓ | ✓ | ✓ | ✓ | ✓ | ✓ | ✓ | ✓ | ✓ | ✓ | ✓ | ✓ | ✓ | Good |
| Jelen et al. (2025)  [39] | ✓ | ✓ | ✓ | ✓ | ✓ | ✓ | ✓ | ✓ | ✓ | ✓ | ✓ | ✓ | ✓ | ✓ | Good |
| Kao et al. (2025)  [42] | ✓ | ✓ | ✓ | ✓ | ✓ | ✓ | ✓ | ✓ | ✓ | ✓ | ✓ | X | ✓ | ✓ | Good |
| Lii et al. (2025)  [40] | ✓ | NR | ✓ | ✓ | ✓ | ✓ | ✓ | ✓ | ✓ | ✓ | ✓ | X | ✓ | ✓ | Good |
| Quintanilla et al. (2023)  [41] | ✓ | NR | ✓ | ✓ | ✓ | ✓ | ✓ | ✓ | ✓ | ✓ | ✓ | X | ✓ | ✓ | Good |
| Saad et al. (2020)  [44] | ✓ | NR | ✓ | ✓ | ✓ | ✓ | ✓ | ✓ | ✓ | ✓ | ✓ | ✓ | ✓ | ✓ | Good |
| Williams et al. (2019)  [37] | ✓ | NR | ✓ | ✓ | ✓ | ✓ | ✓ | ✓ | ✓ | ✓ | ✓ | X | ✓ | ✓ | Good |
| Williams et al. (2018)  [23] | ✓ | NR | ✓ | ✓ | ✓ | ✓ | ✓ | ✓ | ✓ | ✓ | ✓ | X | ✓ | ✓ | Good |
| Yoon et al. (2025)  [38] | ✓ | ✓ | ✓ | ✓ | ✓ | ✓ | ✓ | ✓ | ✓ | ✓ | ✓ | ✓ | ✓ | ✓ | Good |

Symbols: ✓ - yes; X - no

Abbreviations: NR = not reported; NA = not applicable; CD = cannot determine

##

## **Table S3.** Risk of bias/quality assessment of the included studies using the NIH Quality Assessment of Observational Cohort and Cross-Sectional Studies [29,30].

| **Study** | **Item** | | | | | | | | | | | | | | **Quality Rating** |
| --- | --- | --- | --- | --- | --- | --- | --- | --- | --- | --- | --- | --- | --- | --- | --- |
|  | **1** | **2** | **3** | **4** | **5** | **6** | **7** | **8** | **9** | **10** | **11** | **12** | **13** | **14** |  |
| Marton et al. (2019)  [45] | ✓ | ✓ | NA | ✓ | ✓ | ✓ | ✓ | X | ✓ | NR | ✓ | NA | ✓ | X | Fair |

Symbols: ✓ - yes; X - no

Abbreviations: NR = not reported; NA = not applicable; CD = cannot determine

## **Table S4.** Risk of bias/quality assessment of the included studies using the NIH Quality Assessment Tool for Before-After (Pre-Post) Studies with No Control Group [29,30].

| **Study** | **Item** | | | | | | | | | | | | **Quality Rating** |
| --- | --- | --- | --- | --- | --- | --- | --- | --- | --- | --- | --- | --- | --- |
|  | **1** | **2** | **3** | **4** | **5** | **6** | **7** | **8** | **9** | **10** | **11** | **12** |  |
| Yoon et al. (2019)  [47] | ✓ | X | X | NR | X | ✓ | ✓ | X | ✓ | X | ✓ | NA | Poor |

Symbols: ✓ - yes; X - no

Abbreviations: NR = not reported; NA = not applicable; CD = cannot determine

## **Table S5.** Risk of bias/quality assessment of the included studies using the JBI Critical Appraisal Tool for Case Reports [29,31].

| **Study** | **Item** | | | | | | | | **Quality Rating** |
| --- | --- | --- | --- | --- | --- | --- | --- | --- | --- |
|  | **1** | **2** | **3** | **4** | **5** | **6** | **7** | **8** |  |
| Hosanagar et al. (2021)  [46] | ✓ | ✓ | ✓ | ✓ | ✓ | ✓ | ✓ | ✓ | Good |

Symbols: ✓ - yes; X - no

Abbreviations: NR = not reported; NA = not applicable; CD = cannot determine

## **Table S6.** Risk of bias/quality assessment of the included preclinical/animal studies using the SYRCLE’s Risk of Bias tool [32].

| **Study** | **Item** | | | | | | | | | | **Quality Rating** |
| --- | --- | --- | --- | --- | --- | --- | --- | --- | --- | --- | --- |
|  | **1** | **2** | **3** | **4** | **5** | **6** | **7** | **8** | **9** | **10** |  |
| Klein et al. (2020)  [33] | NR | ✓ | NR | NR | NR | NR | NR | ✓ | ✓ | ✓ | Fair |
| Pomrenze et al. (2025)  [34] | ✓ | ✓ | NR | NR | NR | NR | ✓ | ✓ | ✓ | ✓ | Fair |
| Reddy et al. (2021)  [36] | ✓ | ✓ | NR | NR | ✓ | NR | ✓ | ✓ | ✓ | X | Fair |
| Zhang and Hashimoto (2019)  [35] | ✓ | ✓ | NR | NR | NR | NR | NR | ✓ | ✓ | ✓ | Fair |

Symbols: ✓ - yes; X - no

Abbreviations: NR = not reported; NA = not applicable; CD = cannot determine

##

## **Table S7. Hypothesized Roles of Opioid Receptor Subtypes in Depression and Antidepressant Response**

| Receptor Subtype | Summarized Role in Depression and Antidepressant Response |
| --- | --- |
| μ-opioid receptor (MOR) | 1. Regulates reward processing, motivation, and social functioning 2. MOR activation reduces immobility in forced swim and tail suspension test, which is blocked by naloxone (a MOR antagonist) 3. Reduced MOR availability is associated with greater depressive symptoms 4. A118G variant of the MOR gene (OPRM1) is associated with an increase in depression vulnerability |
| δ-opioid receptor (DOR) | 1. The DOR system contributes to reward processing 2. DOR agonists reduce immobility in forced swim test, blocked by naltrindole (a DOR antagonist) 3. DOR agonists may attenuate stress-induced behaviours 4. DOR knockout mice demonstrate pro-depressive behaviours, indicating a role in mood regulation |
| Κ-opioid receptor (KOR) | 1. KOR activation induces pro-depressive-, dysphoric-, anhedonia-, and aversive-like effects 2. Increase in KOR levels are associated with socially stressed humans, potentially linking KOR to depression vulnerability 3. Buprenorphine (a partial MOR agonists and KOR antagonist) reverses anhedonia 4. However, salvinorin A (a KOR agonist) has demonstrated antidepressant-like effects |

*Source*: Jelen LA, et al. Neuroscience & Biobehavioral Reviews. 2022;140:104800. doi: doi:10.1016/j.neubiorev.2022.104800 [25]

##

## **Table S8.** Summary of Heterogeneity Factors Across Preclinical and Clinical Studies on Naltrexone.

| Author(s) | Diagnosis / Model | Sample Size | Intervention | Dose | Route | Dosing Schedule* | Comparator | Measures | Conclusion |
| --- | --- | --- | --- | --- | --- | --- | --- | --- | --- |
| Jelen et al. (2025)  [39] | MDD | 26 | Ketamine + Naltrexone | 0.5mg/kg | Ketamine: IV  Naltrexone: Oral | x1 infusion over 40 minutes | Placebo + Naltrexone | MADRS  QIDS-SR  M3VAS  SHAPS  TEPS  H-fMRS | Mixed Evidence of Opioid Meditation |
| Klein et al. (2020)  [33] | cLH rats | NR | Ketamine | in-vivo: 15mg/kg  ex-vivo: 10μM | IP | x1 injection | Naltrexone | mFST  PRT | Opioid Meditation Supported |
| Marton et al. (2019)  [45] | TRD | 40 | Ketamine | 0.5mg/kg | IV | x2 infusion over 40 minutes weekly  → 3 weeks | Naltrexone | BDI-II | Opioid Meditation NOT Supported |
| Pomrenze et al. (2025)  [34] | CORT mice | NR | Ketamine | 20mg/L | Immersion in water | x1 injection | Naltrexone | FST | Opioid Meditation NOT Supported |
| Williams et al. (2019; 2018)  [23,37] | TRD | 14 | Ketamine + Naltrexone | 0.5mg/kg | Ketamine: IV  Naltrexone: Oral | x1 infusion over 40 minutes | Placebo + Naltrexone | HDRS-17  MADRS  C-SSRS  BDI-II | Opioid Meditation Supported |
| Yoon et al. (2025)  [38] | MDD | 58 | Ketamine + Naltrexone | 0.5mg/kg | Ketamine: IV  Naltrexone: IM | x1 infusion over 40 minutes weekly  → 4 weeks | Placebo + Ketamine  Placebo + Midazolam | MADRS | Opioid Mediation NOT Supported |
| Yoon et al. (2019)  [47] | MDD | 5 | Ketamine | 0.5mg/kg | IV | x1 infusion weekly  → 4 weeks | Naltrexone | MADRS | Opioid Mediation NOT Supported |
| Zhang and Hashimoto (2019)  [35] | CSDS + LPS mice | 80 | Ketamine | 10mg/kg | IP | x1 injection | Naltrexone | FST  TST  SPT | Opioid Mediation NOT Supported |

Abbreviations: BDI-II = Beck Depression Inventory-Second Edition; cLH = congenitally learned helplessness; CORT = corticosterone; CSDS = chronic social defeat stress; C-SSRS = Columbia Suicide Severity Rating Scale; FST = forced swim test; HDRS = Hamilton Depression Rating Scale; H-fMRS = proton functional magnetic resonance spectroscopy; IM = intramuscularly; IP = intraperitoneally; IV = intravenous; LPS = lipopolysaccharide-induced inflammation; M3VAS = Maudsley 3-item Visual Analogue Scale; MADRS = Montgomery-Åsberg Depression Rating Scale; MDD = major depressive disorder; mFST = modified forced swim test; NR = not reported; PRT = progressive ratio task; QIDS-SR = Quick Inventory of Depressive Symptomatology Self-Report; SHAPS = Snaith-Hamilton Pleasure Scale; SPT = sucrose preference test; TEPS = Temporal Experience of Pleasure Scale; TRD = treatment-resistant depression; TST = tail suspension test

* Note: Dosing schedule only refers to ketamine.

## **Table S9.** Summary of Heterogeneity Factors Across Clinical Studies on Opioid Use.

| Author(s) | Diagnosis | Sample Size | Intervention | Dose | Route | Dosing Schedule* | Measures | Comparator | Conclusion |
| --- | --- | --- | --- | --- | --- | --- | --- | --- | --- |
| Hosanagar et al. (2021)  [46] | TRD | 1 | Ketamine | 0.5mg/kg | IV | x2 infusion over 40 minutes weekly  → 4 weeks | MADRS | Buprenorphine | Opioid Mediation NOT Supported |
| Lii et al. (2025)  [40] | MDD | 40 | Ketamine | 0.5mg/kg | IV | x1 infusion over 40 minutes | MADRS  HADS | Placebo | Opioid Mediation NOT Supported |
| Marton et al. (2019)  [45] | TRD | 40 | Ketamine | 0.5mg/kg | IV | x2 infusion over 40 minutes weekly  → 3 weeks | BDI-II | Buprenorphine  Methadone | Opioid Mediation NOT Supported |

Abbreviations: BDI-II = Beck Depression Inventory-Second Edition; HADS = Hospital Anxiety and Depression Scale; IV = intravenous; MADRS = Montgomery-Åsberg Depression Rating Scale; MDD = major depressive disorder; TRD = treatment-resistant depression

* Note: Dosing schedule only refers to ketamine.

## **Table S10.** Summary of Heterogeneity Factors Across Clinical Studies on Opioid Gene Variants.

| Author(s) | Diagnosis | Sample Size | Intervention | Dose | Route | Dosing Schedule* | Comparator | Measures | Gene Variants | Conclusion |
| --- | --- | --- | --- | --- | --- | --- | --- | --- | --- | --- |
| Grunebaum et al. (2020)  [43] | MDD | 71 | Ketamine | 0.5mg/kg | IV | x1 infusion over 40 minutes | Midazolam | HDRS-17  HDRS-24  SSI  POMS | OPRM1: A118G | Opioid Mediation NOT Supported |
| Kao et al. (2025)  [42] | TRD | 65 | Ketamine | 0.2 or 0.5mg/kg | IV | x1 infusion over 40 minutes | Placebo | HADRS  MADRS | - OPRD1  - OPMR1: rs2473546 & rs9479827 | Opioid Mediation Supported |
| Saad et al. (2020)  [44] | TRD | 406 | Esketamine | 84mg | IN | 2x spray weekly  → 4 weeks | Placebo | MADRS | OPRM1:  rs1799971 & rs34427887 | Opioid Mediation NOT Supported |

Abbreviations: HDRS = Hamilton Depression Rating Scale; IN = intranasal; IV = intravenous; MADRS = Montgomery-Åsberg Depression Rating Scale; MDD = major depressive disorder; POMS = Profile of Mood States; SSI = Beck Scale for Suicidal Ideation; TRD = treatment-resistant depression

* Note: Dosing schedule only refers to ketamine.
